# Food and nutrient intake in dietary supplement users: a nationwide school-based study in Japan

**DOI:** 10.1017/jns.2021.96

**Published:** 2022-04-22

**Authors:** Kazue Ishitsuka, Keiko Asakura, Satoshi Sasaki

**Affiliations:** 1 Department of Social and Preventive Epidemiology, Graduate School of Medicine, The University of Tokyo, 7-3-1 Hongo, Bunkyo-ku, Tokyo 113-8654, Japan; 2 Department of Environmental and Occupational Health, School of Medicine, Toho University, Tokyo 143-8540, Japan

**Keywords:** Dietary supplements, School survey, Dietary record, Nutrition epidemiology, BMI, body mass index, CI, confidence interval

## Abstract

Dietary supplements can be used to compensate for an inadequate diet. However, some studies indicate that supplement users consume healthier diets than non-users, although such data are lacking in Japanese children. The aim of the present study was to investigate the food and nutrient intake of dietary supplement users among school-aged children in Japan. A nationwide school dietary survey was conducted at fourteen elementary schools and thirteen junior high schools in twelve prefectures in Japan. Three-day non-consecutive semi-weighed dietary records were obtained on two weekdays and one weekend day. Analysis of covariance was performed to investigate differences in food and nutrient intake from meals consumed at school and outside of school between dietary supplement users and non-users, adjusted for socio-demographic and health-related characteristics. Of the 910 children included in this analysis, 80 (8⋅8 %) reported supplement use. Based on 3-d dietary records, dietary supplement users consumed higher mean amounts of oils and lower amounts of fruits and vegetables than non-users. In school lunches, users consumed significantly higher amounts of oils and lower amounts of protein than non-users. In meals consumed outside of school, dietary supplement users consumed significantly higher amounts of confectioneries and lower amounts of fruits and vegetables, folate, vitamin C and dietary fibre than non-users. Collectively, our findings indicate that dietary supplement users consumed less healthy diets than non-users. Additional studies are warranted to confirm these results and identify factors contributing to poorer dietary habits in supplement users.

## Introduction

Nutrient intake in children and adolescents influences growth and later-life health^(1)^. Research suggests that inadequate intake of calcium and vitamin D during childhood and adolescence increase the risk of low peak bone mass, which is associated with osteoporosis in adulthood^(1)^. Reports have indicated that the intake of certain nutrients, including calcium, iron and vitamin A from food, is insufficient in children, based on dietary reference intake recommendations^(2,3)^. In this regard, dietary supplements may be used to compensate for these deficiencies^(4,5)^.

Some studies suggest that children who are less likely to require dietary supplements may be more likely to use dietary supplements than their counterparts^(6–9)^. The National Health and Nutrition Examination Survey in the USA reported that the intake of calcium, vitamin A, folate and vitamin C was higher in dietary supplement users than in non-users^(6)^. Studies in Korea have also demonstrated that the intake of calcium, vitamin A and folate was higher in dietary supplement users than in non-users^(7,8)^. Similarly, a study in Puerto Rico reported that the intake of calcium and folate was higher in dietary supplement users than in non-users^(9)^. Furthermore, a study in the USA demonstrated that the intake of vegetables and fruits was higher and the intake of saturated fats was lower in dietary supplement users than in non-users, suggesting that dietary supplement users consumed healthier diets than non-users^(10)^. The guardians of children who use dietary supplements may be more likely to practice health-conscious behaviours that are passed on to their children.

Dietary habits and dietary supplement use are population-specific and are influenced by sociocultural factors and the availability of food and dietary supplements^(11)^. To date, no study has examined the association between dietary supplement use and dietary intake in Japan. The Japanese diet is considered to be healthy in terms of high intake of vegetables, seaweed, soyabeans, fish and low intake of processed meat and soft drink^(11–13)^. Given that the prevalence of dietary supplement use is relatively low in Japan^(5,14)^, the dietary habits of Japanese dietary supplement users may differ from those in countries with a high prevalence of dietary supplement use^(7,15,16)^. Furthermore, 99 % of elementary schools and 89 % of junior high schools provide school lunches to all students in Japan^(17)^, with a standardised menu provided to all children at any given school. As such, school lunches are unlikely to reflect health-conscious practices of guardians, whereas other meals may be more reflective of these habits.

Here, the present study aimed to investigate whether dietary supplement users had healthier diets than non-users among school-aged children in Japan. To this end, we investigated differences in food and nutrient intake from foods between users and non-users of dietary supplements among Japanese children in elementary and junior high schools. Furthermore, we investigated food and nutrient intake from foods consumed in school lunches and meals outside of school between users and non-users of dietary supplements, respectively.

## Methods

### Study design and participants

**Fig. 1.**
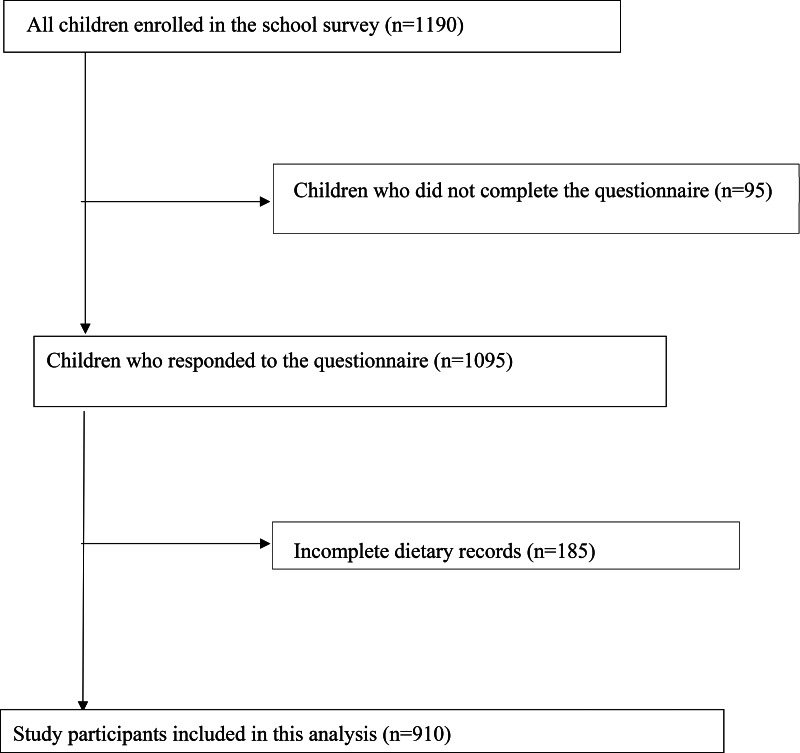
Flow diagram of study participants.

The present study employed a cross-sectional school survey supported by the Ministry of Education, Culture, Sports, Science and Technology of Japan and local educational boards at the prefectural and municipal levels^(3,18)^. The design of the school survey has been described previously in detail^(3)^. Briefly, the school survey aimed to investigate the dietary intake of children in elementary and junior high schools and was conducted periodically. Eligible criteria for this survey were children in grades 3 (8–9 years old) and 5 (10–11 years old) in elementary school and children in grade 8 (13–14 years old) in junior high school. Children were recruited from fourteen elementary schools and thirteen junior high schools in twelve prefectures encompassing a wide geographic area of Japan from November to December 2014. Nutrition teachers were involved in dietary assessment as dietitians. One class comprising a minimum of thirty children was recruited from each school. A total of 1190 students participated in the survey. A flow diagram of the study participants is presented in Fig. 1. In total, 910 children who completed 3-d dietary records and a questionnaire on dietary supplement use were included in the final analysis.

The study protocol was reviewed and approved by the University of Tokyo (#10653). The study was conducted according to the guidelines of the Declaration of Helsinki. Written informed consent was obtained from the guardians of all participating children.

### Measurement of dietary supplement use and dietary intake

For the present study, dietary supplements were defined as containing at least one vitamin and one mineral because earlier studies indicated that vitamin and mineral supplements are associated with adequate nutrient intake from food^(6,9)^. Children were required to indicate the frequency of dietary supplement use in the preceding month, including tablets containing vitamins, calcium, iron and other supplement ingredients. Guardians assisted their children in answering the survey questions. Children were required to choose from the following options: twice a day, once a day, 4–6 times/week, 2–3 times/week, once a week, less than once a week and never. Dietary supplement users were defined as school children who used dietary supplements at least once in the preceding month^(4)^.

Dietary intake from food was assessed using 3-d, non-consecutive semi-weighed dietary records. The 3 days consisted of two weekdays on which school lunches were provided and one weekend day on which school lunches were not provided. On weekdays, school dietitians performed dietary records for school lunches on days without special events, and on the same day, guardians who prepared food for their children (predominantly mothers) performed dietary records for meals outside of school. For each child, school dietitians assessed dietary intake from school lunches by first weighing the ingredients in all dishes before cooking; weighing the cooked foods and, finally, weighing any leftover food. Guardians were provided instruction manuals for completing dietary records. School dietitians checked dietary records completed by guardians. Finally, all dietary records were assessed by research dietitians in the research office at the university. Two research dietitians verified the recorded food items and weights at the research office.

### Calculation of food and nutrient intake

The intake of energy and nutrients was estimated using the Standard Tables of Food Composition in Japan^(19)^. The amounts of food and nutrients consumed were energy-adjusted using the density method to mitigate any misreporting associated with dietary assessment and biases due to variations in body size and energy requirements^(20)^. Based on similarities in nutrient profiles and culinary habits, foods were categorised into the following food groups: (1) cereals, (2) potatoes, (3) confectionaries, (4) nuts and pulses, (5) fruits and vegetables, (6) oils, (7) teas, (8) fish, (9) meats and eggs, (10) dairy products and (11) seasonings (Supplementary Table S1)^(19)^. We also assessed the intake of the following nutrients: calcium, iron, vitamin A, vitamin D, folate, vitamin C, protein, *n*–3 polyunsaturated fat, saturated fat and dietary fibre, given that these nutrients are often present in dietary supplements and are potentially linked to the health-consciousness of guardians^(6,7,10,15,16)^. All nutrient intake was estimated based on only food intake. To assess misreporting of dietary intake, we calculated the ratio of energy intake based on dietary records to estimated energy requirements derived from Japanese dietary reference intake recommendations^(21)^.

### Measurement of other variables

Height and weight were measured by school nurses during school health check-ups. The body mass index (BMI) of children was categorised as underweight, normal-weight or overweight using the age- and sex-specific cut-off points defined by the International Obesity Task Force^(22,23)^. In adults, these values are BMI < 18⋅5 kg/m^2^ for underweight, 18⋅5–24⋅9 kg/m^2^ for normal-weight and ≥25 kg/m^2^ for overweight or obese^(22,23)^.

Children reported on their sports participation based on whether they participated in sports clubs and/or other physical activities in the previous month. The frequency of sports participation was reported by selecting from the following responses: never, once a week, 2–3 times/week, 4–6 times/week and every day.

Guardians completed questionnaires about their employment status and concerns about their child's health and nutrition (picky eater, small appetite or overeating). Guardians’ employment status was categorised as unemployed, part-time worker (<40 h/week) or full-time worker (≥40 h/week).

Given that previous reports have indicated an association between fruit and vegetable intake and dietary supplement use^(10,24–29)^, we investigated potential factors associated with fruit and vegetable intake, including guardian's influence on fruit and vegetable intake and perceived home food environment^(30–32)^ using the following questions: (1) ‘Do you intend to provide your child with fruits and vegetables?’ (intention to provide children with fruits and vegetables); (2) ‘Do you think the place where you obtain fruits and vegetables is convenient?’ (accessibility to fruits and vegetables); (3) ‘Are you satisfied with the quality of fruits and vegetables that are available near your house?’ (satisfaction with the quality of fruits and vegetables available); (4) ‘Are you satisfied with the variety of fruits and vegetables at the supermarket near your house?’ (satisfaction with the variety of fruits and vegetables available) and (5) ‘Do you think fruits and vegetables are too expensive?’ (affordability of fruits and vegetables). Guardians selected from the following response options: strongly agree, agree, do not agree, do not agree at all and not sure. For analysis, the response options were stratified into agree (strongly agree and agree) or do not agree (do not agree, do not agree at all and not sure).

Income per capita was defined as the mean income of the municipality in which the participant lived. Income per capita is characterised by community-level socioeconomic status, whereas household income is characterised by household-level socioeconomic status. Information on income per capita was determined based on school location. Income per capita (Japanese yen) was calculated using the census tracts for municipalities^(33)^, as follows: income per capita = (aggregated annual taxable income)/(total municipal population). Residential areas were divided into three categories according to population size based on the National Health and Nutrition Survey: <50 000, 50 000–150 000 and ≥150 000^(34)^.

### Statistical analysis

Dietary supplement users were defined as children who used dietary supplements at least once in the preceding month. Characteristics of dietary supplement users and non-users were compared using *P* for trend. Analysis of covariance was performed to investigate differences in food and nutrient intake between users and non-users. Age, sex, sports participation, BMI, guardian's employment status, population size of residence area and income per capita were used as covariates in multivariate analysis^(7,9,10,15,35–41)^. We calculated crude and adjusted mean intake of food, energy and nutrients from all meals derived from 3-d dietary records for two weekdays and one weekend day using least-square means from analysis of covariance. We also calculated crude and adjusted mean intake of food, energy and nutrients from school lunches and meals consumed outside of school using 2-d dietary records for weekdays.

Multivariate linear regression analyses were subsequently conducted to investigate the effects of the food environment on the association between dietary supplement use and the intake of fruits and vegetables from meals consumed outside of school using 2-d dietary records for weekdays. Given that each indicator of the home food environment (accessibility, satisfaction with quality, satisfaction with variety and affordability) may be independently associated with fruit and vegetable intake as well as with each other, each indicator was examined in a separate regression model. Five models were developed for the analysis: model 1 was adjusted for age, sex, sports participation, BMI, guardian's employment status, population size of residence area and income per capita; model 2 was adjusted for accessibility to fruits and vegetables and covariates used in model 1; model 3 was adjusted for satisfaction with the quality of fruits and vegetables and covariates used in model 1; model 4 was adjusted for satisfaction with the variety of fruits and vegetables and covariates used in model 1 and model 5 was adjusted for the affordability of fruits and vegetables and covariates used in model 1. Coefficients for linear regression models and 95 % confidence intervals (CIs) were calculated.

Given that the present study involved exploratory analyses using secondary data, sample size calculation was not performed. All statistical analyses were conducted using SAS version 9.4 (SAS Institute, Cary, NC, USA). *P* < 0⋅05 were considered statistically significant.

## Results

Of the 910 children included in the present analysis, eighty (8⋅8 %) used dietary supplements and twenty (2⋅3 %) used dietary supplements every day. The mean (standard deviation) ratio of energy intake from dietary records to estimated energy requirements based on Japanese dietary reference intake recommendations was 1⋅02 (0⋅20).

Table 1 shows the characteristics of the study participants grouped by users and non-users of dietary supplements. Dietary supplement users were significantly older and participated less frequently in sports than non-users. Income per capita was significantly higher for dietary supplement users than for non-users. Most guardians reported that they intended to provide fruits and vegetables to their children. Accessibility to fruits and vegetables was significantly lower for dietary supplement users than for non-users (49 [64⋅5 %] *v*. 689 [84⋅3 %], *P* < 0⋅05). Satisfaction with the variety of fruits and vegetables available was also significantly lower in dietary supplement users than in non-users (54 [70⋅1 %] *v*. 656 [80⋅4 %], *P* < 0⋅05).

**Table 1.** Characteristics of children and their guardians grouped by users and non-users of dietary supplements*

Characteristic	Users of dietary supplements (*n* 80)		Non-users of dietary supplements (*n* 830)		
	*n*	(%)	*n*	(%)	*P*†
Age					0⋅0235
8–9 years old (3rd grade)	22	(27⋅5)	287	(34⋅6)	
10–11 years old (5th grade)	23	(28⋅8)	298	(35⋅9)	
13–14 years old (8th grade)	35	(43⋅8)	245	(29⋅5)	
Sex					0⋅0600
Female	34	(42⋅5)	444	(53⋅5)	
Male	46	(57⋅5)	386	(46⋅5)	
BMI category‡					0⋅2644
Underweight	15	(18⋅8)	164	(19⋅8)	
Normal-weight	61	(76⋅3)	611	(73⋅6)	
Overweight/obese	4	(5⋅0)	55	(6⋅6)	
Frequency of sports participation					0⋅0008
Never	31	(38⋅8)	191	(23⋅0)	
Once a week	16	(20⋅0)	159	(19⋅2)	
2–3 per week	18	(22⋅5)	192	(23⋅1)	
4–6 per week	4	(5⋅0)	122	(14⋅7)	
Every day	9	(11⋅3)	146	(17⋅6)	
Missing data	2	(2⋅5)	20	(2⋅4)	
Guardian's employment status					0⋅2832
Unemployed	10	(12⋅5)	160	(19⋅3)	
Part-time worker	37	(46⋅3)	345	(41⋅6)	
Full-time worker	29	(36⋅3)	283	(34⋅1)	
Missing data	4	(5⋅0)	42	(5⋅1)	
Income per capita					0⋅0025
First quartile	12	(15⋅0)	231	(27⋅8)	
Second quartile	11	(13⋅8)	181	(21⋅8)	
Third quartile	32	(40⋅0)	213	(25⋅7)	
Fourth quartile	25	(31⋅3)	205	(24⋅7)	
Population size of residence area at municipality levels					0⋅0735
<50 000	31	(38⋅8)	402	(48⋅4)	
50 000–150 000	14	(17⋅5)	84	(10⋅1)	
≥150 000	35	(43⋅8)	344	(41⋅5)	
Guardian's concerns about child's health and nutrition					
Picky eater					0⋅5799
Yes	27	(33⋅8)	271	(32⋅7)	
No	45	(56⋅3)	520	(62⋅7)	
Missing data	8	(10⋅0)	39	(4⋅7)	
Small appetite					0⋅3125
Yes	15	(18⋅8)	131	(15⋅8)	
No	55	(68⋅8)	654	(78⋅8)	
Missing data	10	(12⋅5)	45	(5⋅4)	
Overeating					0⋅3375
Yes	7	(8⋅8)	112	(13⋅5)	
No	62	(77⋅5)	670	(80⋅7)	
Missing data	11	(13⋅8)	48	(5⋅8)	
Guardian's intention to provide children with fruits and vegetables					0⋅5296
Agree	77	(100⋅0)	806	(99⋅1)	
Do not agree	0	(0⋅0)	7	(0⋅9)	
Missing data	3	(3⋅8)	17	(2⋅1)	
Guardian's perception of home food environment§					
Accessibility to fruits and vegetables					<0⋅001
Agree	49	(64⋅5)	689	(84⋅3)	
Do not agree	27	(35⋅5)	128	(15⋅7)	
Missing data	4	(5⋅0)	13	(1⋅6)	
Satisfaction with the quality of fruits and vegetables available					0⋅0330
Agree	54	(70⋅1)	656	(80⋅4)	
Do not agree	23	(29⋅9)	160	(19⋅6)	
Missing data	3	(3⋅8)	14	(1⋅7)	
Satisfaction with the variety of fruits and vegetables available					0⋅3628
Agree	55	(72⋅4)	629	(77⋅0)	
Do not agree	21	(27⋅6)	188	(23⋅0)	
Missing data	4	(5⋅0)	13	(1⋅6)	
Affordability of fruits and vegetables					0⋅5545
Agree	55	(71⋅4)	608	(74⋅5)	
Do not agree	22	(28⋅6)	208	(25⋅5)	
Missing data	3	(3⋅8)	14	(1⋅7)	

BMI, body mass index.

* Non-users were defined as children and adolescents who did not use dietary supplements in the preceding month.

† *P* for trend was assessed after removing missing data.

‡ Child BMI was categorised as underweight, normal-weight or overweight based on age- and sex-specific cut-off points defined by the International Obesity Task Force^(22,23)^.

§ Guardian's perception of the home food environment was assessed using the following questions: (1) ‘Do you think the place where you obtain fruits and vegetables is convenient?’ (accessibility to fruits and vegetables); (2) ‘Are you satisfied with the quality of fruits and vegetables that are available near your house?’ (satisfaction with the quality of fruits and vegetables available); (3) ‘Are you satisfied with the variety of fruits and vegetables at the supermarket near your house?’ (satisfaction with the variety of fruits and vegetables available) and (4) ‘Do you think fruits and vegetables are too expensive?’ (affordability of fruits and vegetables).

Table 2 shows the mean food, energy and nutrient intake of dietary supplement users and non-users based on 3-d records of all meals consumed on weekdays and a weekend day. Dietary supplement users had a significantly lower intake of fruits and vegetables than non-users (adjusted mean: 135⋅1 *v*. 150⋅7 g/1000 kcal, *P* < 0⋅05).

**Table 2.** Food, energy and nutrient intake grouped by users and non-users of dietary supplements based on 3-d records*

	Users of dietary supplements			Non-users of dietary supplements			
	Mean	sd	Adjusted mean†	Mean	sd	Adjusted mean†	*P*†
Food group							
Cereals (g/1000 kcal)	227⋅8	48⋅5	225⋅9	227⋅8	42⋅9	232⋅0	0⋅2377
Potatoes (g/1000 kcal)	23⋅4	15⋅1	22⋅9	25⋅1	16⋅9	24⋅6	0⋅4352
Confectionaries (g/1000 kcal)	29⋅5	19⋅1	28⋅0	27⋅8	19⋅8	25⋅7	0⋅3391
Nuts and pulses (g/1000 kcal)	23⋅8	18⋅4	22⋅9	27⋅0	18⋅0	24⋅9	0⋅3479
Fruits and vegetables (g/1000 kcal)	137⋅9	58⋅8	135⋅1	156	58⋅4	150⋅7	0⋅0281
Oils (g/1000 kcal)	8⋅1	4⋅8	8⋅0	7⋅1	3⋅6	7⋅3	0⋅1142
Teas (g/1000 kcal)	81⋅1	88⋅5	85⋅4	104⋅2	114⋅6	103⋅9	0⋅1878
Fish (g/1000 kcal)	67⋅3	50⋅3	70⋅0	70⋅4	50⋅8	73⋅1	0⋅6293
Meats and eggs (g/1000 kcal)	129⋅5	61⋅9	94⋅9	122⋅4	51⋅7	97⋅3	0⋅6867
Dairy products (g/1000 kcal)	120⋅3	55⋅5	133⋅0	116⋅7	53⋅8	121⋅1	0⋅0683
Seasonings (g/1000 kcal)	47⋅9	34⋅0	47⋅1	52⋅4	35⋅9	53⋅3	0⋅1467
Energy (kcal/day)	2274	568	2142	2097	448	2102	0⋅3860
Nutrient							
Calcium (mg/1000 kcal)	295	67	302	302	68	298	0⋅5869
Iron (mg/1000 kcal)	3⋅3	0⋅6	3⋅3	3⋅5	0⋅6	3⋅4	0⋅1004
Vitamin A (μgRE/1000 kcal)	245	86	243	249	88	241	0⋅8422
Vitamin D (mg/1000 kcal)	3⋅3	2⋅2	3⋅4	2⋅9	2⋅0	3⋅0	0⋅1618
Folate (μg/1000 kcal)	127	33	125	137	37	133	0⋅0798
Vitamin C (mg/1000 kcal)	41	18	40	45	19	44	0⋅1054
Protein (g/1000 kcal)	35⋅4	4⋅3	35⋅2	36⋅2	4⋅1	35⋅8	0⋅2744
*n*–3 polyunsaturated fat (% energy)	0⋅9	0⋅3	0⋅9	0⋅9	0⋅3	0⋅9	0⋅6592
Saturated fatty acid (% energy)	2⋅1	9⋅9	10⋅1	9⋅9	1⋅9	9⋅7	0⋅1016
Dietary fibre (g/1000 kcal)	6⋅0	1⋅7	5⋅8	6⋅4	1⋅5	6⋅1	0⋅0739

sd, standard deviation

* Food and nutrient intake per day was calculated using 3-d dietary records. Nutrient intake was estimated based on foods only, and nutrient intake from dietary supplements was not included.

† Analysis of covariance adjusted for age, sex, body mass index, sports participation, guardian's employment status, population size of residence area and income was performed.

Table 3 shows the food, energy and nutrient intake from school lunches and meals consumed outside of school among dietary supplement users and non-users based on 2-d records of meals consumed on weekdays. In school lunches, dietary supplement users had a significantly higher intake of energy (adjusted mean 775 *v*. 732 kcal, *P* < 0⋅05) and oils (7⋅3 *v*. 5⋅7 g/1000 kcal, *P* < 0⋅05) and a significantly lower intake of protein (36⋅2 *v*. 37⋅2 g/1000 kcal, *P* < 0⋅05) than non-users. In meals consumed outside of school, dietary supplement users had a significantly higher intake of confectioneries (42⋅3 *v*. 47⋅2 g/1000 kcal, *P* < 0⋅05) and a significantly lower intake of fruits and vegetables (130⋅9 *v*. 227⋅4 g/1000 kcal, *P* < 0⋅05), folate (125 *v*. 140 μg/1000 kcal, *P* < 0⋅05), vitamin C (41 *v*. 50 mg/1000 kcal, *P* < 0⋅05) and dietary fibre (5⋅5 *v*. 6⋅1 g/1000 kcal, *P* < 0⋅05).

**Table 3.** Food, energy and nutrient intake grouped by users and non-users of dietary supplements based on 2-d records on school days*

	School lunch							Other than school lunch						
	Users of dietary supplements			Non-users of dietary supplements				Users of dietary supplements			Non-users of dietary supplements			
	Mean	sd	Adjusted mean†	Mean	sd	Adjusted mean†	*P*†	Mean	sd	Adjusted mean†	Mean	sd	Adjusted mean†	*P*†
Food group														
Cereals (g/1000 kcal)	207⋅0	81⋅4	262⋅3	178⋅6	62⋅4	258⋅5	0⋅3449	205⋅6	66⋅5	202⋅5	213⋅2	62⋅4	213⋅3	0⋅1099
Potatoes (g/1000 kcal)	21⋅1	11⋅3	25⋅6	21⋅2	11⋅9	27⋅8	0⋅3226	22⋅3	23⋅6	22⋅9	24⋅9	27⋅3	26⋅8	0⋅3910
Confectionaries (g/1000 kcal)	3⋅8	6⋅4	4⋅6	5⋅0	7⋅6	6⋅3	0⋅1954	44⋅7	32⋅4	42⋅3	35⋅2	31⋅5	47⋅2	0⋅0220
Nuts and pulses (g/1000 kcal)	23⋅8	22⋅3	30⋅2	28⋅8	26⋅3	34⋅0	0⋅1767	24⋅3	30⋅4	25⋅4	25⋅4	26⋅7	34⋅1	0⋅9427
Fruits and vegetables (g/1000 kcal)	147⋅9	51⋅7	158⋅0	137⋅5	57⋅3	170⋅3	0⋅1542	122⋅3	75⋅0	130⋅9	159⋅5	86⋅9	227⋅4	0⋅0071
Oils (g/1000 kcal)	7⋅0	10⋅2	7⋅3	4⋅1	4⋅6	5⋅7	0⋅0314	7⋅6	5⋅8	7⋅2	7⋅6	5⋅5	11⋅0	0⋅9200
Teas (g/1000 kcal)	0⋅0	0⋅0	0⋅0	0⋅0	0⋅0	0⋅0	-	113⋅9	134⋅4	130⋅1	143⋅9	166⋅4	145⋅0	0⋅3343
Fish (g/1000 kcal)	37⋅6	27⋅7	38⋅0	29	24⋅9	37⋅5	0⋅9703	51⋅8	55⋅8	55⋅6	52⋅1	54⋅9	53⋅8	0⋅7620
Meats and eggs (g/1000 kcal)	25⋅1	13⋅8	32⋅3	22⋅8	13⋅2	31⋅5	0⋅4751	62⋅6	35⋅2	39⋅2	67⋅8	36⋅5	43⋅3	0⋅2957
Dairy products (g/1000 kcal)	220⋅1	27⋅6	297⋅6	210⋅5	38⋅9	298⋅4	0⋅9744	65	75⋅5	75⋅5	66⋅3	77⋅1	72⋅9	0⋅5494
Seasonings (g/1000 kcal)	53⋅7	65⋅5	70⋅9	44⋅6	53⋅5	78⋅8	0⋅3547	37⋅7	50⋅7	37⋅0	47⋅6	51⋅4	41⋅9	0⋅1550
Energy (kcal/meal)	818	211	775	729	158	732	0⋅0152	1557	471	1488	1426	385	1444	0⋅4030
Nutrient														
Calcium (mg/1000 kcal)	477	87	470	504	89	480	0⋅2914	238	100	254	249	98	251	0⋅8374
Iron (mg/1000 kcal)	3⋅2	0⋅7	3⋅0	3⋅4	0⋅7	3⋅1	0⋅0983	3⋅4	0⋅8	3⋅4	3⋅6	0⋅9	3⋅5	0⋅1572
Vitamin A (μgRE/1000 kcal)	338	99	305	357	128	319	0⋅1900	224	151	234	234	152	235	0⋅9500
Vitamin D (mg/1000 kcal)	3⋅7	3⋅1	3⋅4	3⋅2	3⋅0	3⋅1	0⋅2744	3⋅4	3⋅7	3⋅5	3⋅0	3⋅3	3⋅3	0⋅3786
Folate (μg/1000 kcal)	146	39	124	152	50	131	0⋅1551	119	46	125	139	54	140	0⋅0278
Vitamin C (mg/1000 kcal)	41	16	34	43	24	36	0⋅3329	40	26	41	48	28	50	0⋅0187
Protein (g/1000 kcal)	37⋅7	4⋅6	36⋅2	38⋅8	3⋅7	37⋅2	0⋅0200	34⋅8	6⋅3	35⋅1	35⋅9	6⋅2	14⋅2	0⋅3680
*n*–3 polyunsaturated fat (% energy)	0⋅7	0⋅5	0⋅6	0⋅6	0⋅3	0⋅6	0⋅6027	1⋅0	0⋅6	1⋅0	0⋅9	0⋅5	0⋅9	0⋅1252
Saturated fatty acid (% energy)	10⋅5	2⋅0	10⋅2	10⋅7	1⋅9	10⋅2	0⋅8871	10⋅1	3⋅35	10⋅2	9⋅6	2⋅9	9⋅6	0⋅0840
Dietary fibre (g/1000 kcal)	7⋅8	4⋅0	6⋅5	7⋅8	3⋅0	6⋅6	0⋅7333	5⋅4	1⋅9	5⋅5	6⋅2	2⋅0	6⋅1	0⋅0041

sd, standard deviation

* Food and nutrient intake on school days was calculated using 2-d dietary records. Nutrient intake was estimated based on foods only, and nutrient intake from dietary supplements was not included.

† Analysis of covariance adjusted for age, sex, body mass index, sports participation, guardian's employment status, population size of residence area and income was performed.

Table 4 shows the associations between dietary supplement use and fruit and vegetable intake at meals consumed outside of school after adjusting for perceived home food environment. In model 1 (adjusted for child age, sex, BMI, sports participation, guardian's employment status and income), dietary supplement use was significantly associated with a lower intake of fruits and vegetables (coefficient: −29⋅3, 95 % CI −50⋅6, −8⋅0, *P* < 0⋅05). Further adjustment for each indicator of home food environment (accessibility, satisfaction with quality, satisfaction with variety and affordability) in addition to the covariates in model 1 attenuated the regression coefficient in each model (model 2, −24⋅8 [95 % CI −46⋅5, −3⋅1]; model 3, −27⋅7 [95 % CI −49⋅2, −6⋅3]; model 4, −27⋅1 [95 % CI −48⋅7, −5⋅6] and model 5, −28⋅5 [95 % CI −50⋅0, −7⋅1]).

**Table 4.** Multiple linear regression models of the association between dietary supplement use and fruit and vegetable intake outside of school*

	Fruit and vegetable intake																			
	Model 1				Model 2				Model 3				Model 4				Model 5			
	*β*	(95 % CI)		*P*†	*β*	(95 % CI)		*P*†	*β*	(95 % CI)		*P*†	*β*	(95 % CI)		*P*†	*β*	(95 % CI)		*P*†
Dietary supplement use																				
Users	−29⋅3	−50⋅6	−8⋅0	0⋅0071	−24⋅8	−46⋅5	−3⋅1	0⋅0248	−27⋅7	−49⋅2	−6⋅3	0⋅0114	−27⋅1	−48⋅7	−5⋅6	0⋅0136	−28⋅5	−50⋅0	−7⋅1	0⋅0092
Non−users	Ref				Ref				Ref											
Age (years old)																				
8–9	Ref				Ref				Ref				Ref				Ref			
10–11	11⋅6	−2⋅9	26⋅1	0⋅1172	11⋅8	−2⋅8	26⋅3	0⋅1125	11⋅8	−2⋅8	26⋅4	0⋅1131	11⋅8	−2⋅8	26⋅4	0⋅1122	11⋅9	−2⋅7	26⋅5	0⋅1091
13–14	14⋅3	−3⋅4	32⋅0	0⋅1130	17⋅3	−0⋅4	35⋅1	0⋅0558	14⋅5	−3⋅2	32⋅3	0⋅1073	14⋅8	−2⋅9	32⋅5	0⋅1012	13⋅4	−4⋅4	31⋅2	0⋅1400
Sex																				
Male	Ref				Ref				Ref				Ref				Ref			
Female	16⋅1	4⋅0	28⋅3	0⋅0094	15⋅8	3⋅7	28⋅0	0⋅0107	16⋅2	4⋅0	28⋅4	0⋅0094	15⋅8	3⋅6	28⋅0	0⋅0112	15⋅8	3⋅6	28⋅0	0⋅0114
BMI category‡																				
Underweight	−5⋅8	−27⋅4	15⋅8	0⋅6001	−4⋅8	−26⋅4	16⋅7	0⋅6613	−4⋅8	−26⋅6	16⋅9	0⋅6624	−5⋅1	−26⋅7	16⋅5	0⋅6416	−5⋅9	−27⋅5	15⋅8	0⋅5939
Normal-weight	Ref				Ref				Ref				Ref				Ref			
Overweight/obese	−4⋅8	−20⋅1	10⋅6	0⋅5424	−5⋅3	−20⋅7	10⋅1	0⋅4965	−4⋅8	−20⋅2	10⋅6	0⋅5415	−4⋅9	−20⋅3	10⋅5	0⋅5297	−4⋅2	−19⋅6	11⋅2	0⋅5924
Frequency of sports participation																				
Never	Ref				Ref				Ref				Ref				Ref			
Once a week	22⋅7	4⋅1	41⋅4	0⋅0170	22⋅2	3⋅6	40⋅9	0⋅0194	22⋅4	3⋅7	41⋅1	0⋅0188	22⋅4	3⋅7	41⋅1	0⋅0190	22⋅9	4⋅1	41⋅6	0⋅0167
2–3 per week	25⋅2	5⋅5	45⋅0	0⋅0124	24⋅2	4⋅4	43⋅9	0⋅0165	24⋅7	4⋅9	44⋅5	0⋅0145	24⋅7	5⋅0	44⋅5	0⋅0142	24⋅5	4⋅7	44⋅4	0⋅0156
4–6 per week	28⋅2	6⋅2	50⋅2	0⋅0120	28⋅8	6⋅8	50⋅7	0⋅0102	28⋅0	6⋅0	50⋅0	0⋅0127	28⋅1	6⋅1	50⋅0	0⋅0125	28⋅4	6⋅3	50⋅4	0⋅0117
Every day	23⋅1	2⋅1	44⋅1	0⋅0312	21⋅6	0⋅6	42⋅6	0⋅0439	22⋅5	1⋅4	43⋅5	0⋅0364	23⋅0	2⋅0	44⋅0	0⋅0320	22⋅8	1⋅7	43⋅9	0⋅0340
Guardian's employment status																				
Unemployed	Ref				Ref				Ref				Ref				Ref			
Part-time worker	−7⋅0	−23⋅1	9⋅2	0⋅3988	−7⋅9	−24⋅0	8⋅3	0⋅3396	−6⋅9	−23⋅1	9⋅3	0⋅4040	−6⋅7	−22⋅9	9⋅5	0⋅4177	−6⋅6	−22⋅8	9⋅6	0⋅4257
Full-time worker	−6⋅5	−23⋅3	10⋅2	0⋅4438	−6⋅4	−23⋅1	10⋅4	0⋅4568	−6⋅4	−23⋅2	10⋅4	0⋅4546	−5⋅9	−22⋅7	10⋅9	0⋅4917	−6⋅1	−22⋅9	10⋅7	0⋅4787
Income per capita																				
First quartile	Ref				Ref				Ref				Ref				Ref			
Second quartile	−0⋅4	−19⋅4	18⋅5	0⋅9630	−1⋅8	−20⋅8	17⋅2	0⋅8546	−2⋅5	−21⋅6	16⋅6	0⋅7986	−3⋅4	−22⋅5	15⋅7	0⋅7273	−1⋅7	−20⋅7	17⋅4	0⋅8649
Third quartile	3⋅1	−17⋅5	23⋅7	0⋅7684	0⋅9	−19⋅7	21⋅5	0⋅9318	1⋅2	−19⋅6	22⋅0	0⋅9101	1⋅0	−19⋅8	21⋅8	0⋅9254	2⋅0	−18⋅7	22⋅8	0⋅8490
Fourth quartile	0⋅4	−24⋅1	25⋅0	0⋅9725	0⋅6	−23⋅9	25⋅2	0⋅9594	−1⋅6	−26⋅3	23⋅1	0⋅8993	−3⋅7	−28⋅5	21⋅1	0⋅7705	−1⋅0	−25⋅8	23⋅7	0⋅9339
Population size of residence area																				
<50 000	Ref				Ref				Ref				Ref				Ref			
50 000–150 000	−8⋅5	−32⋅9	15⋅9	0⋅4926	−8⋅0	−32⋅4	16⋅4	0⋅5218	−8⋅4	−32⋅8	16⋅1	0⋅5023	−9⋅1	−33⋅6	15⋅3	0⋅4631	−10⋅3	−34⋅8	14⋅3	0⋅4129
≥150 000	−10⋅0	−28⋅9	8⋅9	0⋅3000	−11⋅7	−30⋅6	7⋅3	0⋅2260	−10⋅2	−29⋅2	8⋅7	0⋅2891	−9⋅7	−28⋅7	9⋅3	0⋅3150	−9⋅8	−28⋅7	9⋅2	0⋅3119
Accessibility to fruits and vegetables																				
Yes					24⋅6	8⋅7	40⋅6	0⋅0025												
No					Ref															
Satisfaction with quality of fruits and vegetables																				
Yes									14⋅1	−0⋅6	28⋅9	0⋅0600								
No									Ref											
Satisfaction with the variety of fruits and vegetables																				
Yes													14⋅8	0⋅6	28⋅9	0⋅0406				
No													Ref							
Affordability of fruits and vegetables																				
Yes																	−9⋅2	−22⋅9	4⋅5	0⋅1891
No																	Ref			

*β*, coefficient of regression model; BMI, body mass index; CI, confidence interval; Ref, reference.

* The intake of fruits and vegetables in all meals except for school lunches was estimated using 2-d dietary records on weekdays. The explanatory variable was dietary supplement use.

† Five models were developed for the analysis: model 1 was adjusted for age, sex, sports participation, BMI, guardian's employment status, population size of residence area and income per capita; model 2 was adjusted for accessibility to fruits and vegetables and covariates used in model 1; model 3 was adjusted for satisfaction with the quality of fruits and vegetables and covariates used in model 1; model 4 was adjusted for satisfaction with the variety of fruits and vegetables and covariates used in model 1 and model 5 was adjusted for the affordability of fruits and vegetables and covariates used in model 1.

‡ Child BMI was categorised as underweight, normal-weight or overweight based on age- and sex-specific cut-off points defined by the International Obesity Task Force^(22,23)^.

## Discussion

Evidence suggests that dietary supplement users may consume healthier diets than non-users, but there is a lack of data in the Japanese population. This is the first study to investigate the association between dietary supplement use and dietary intake in Japanese children. Data from 3-d dietary records indicated that dietary supplement users had a higher intake of oils and a lower mean intake of fruits and vegetables than non-users. In school lunches, dietary supplement users had a significantly higher intake of oils and a significantly lower intake of protein than non-users. In meals consumed outside of school lunches, dietary supplement users had a significantly higher intake of confectioneries and a significantly lower intake of fruits and vegetables, folate, vitamin C and dietary fibre.

Few studies have examined the associations between dietary supplement use and food intake in children. A study in the USA reported that the intake of fruits and vegetables was significantly higher and the intake of fried foods and soft drinks was significantly lower in dietary supplement users than in non-users, suggesting that dietary supplement users consumed healthier diets than non-users^(10)^ Studies on adults in the USA and European countries revealed that fruit and vegetable intake was significantly higher in dietary supplement users than in non-users^(24–30)^. This may be explained by the fact that dietary supplement users are more likely to be health-conscious than non-users^(42)^. In this regard, the guardians of children who use dietary supplements may be more likely to practice health-conscious behaviours that are assimilated by their children.

In contrast to previous findings, we observed that fruit and vegetable intake was significantly lower and confectionary intake was significantly higher in dietary supplement users than in non-users, suggesting that dietary supplement users were less likely to consume healthy diets than non-users in the present study for Japanese children. Our findings are inconsistent with the results of previous studies^(6,7,15,16)^. To date, there have been no studies investigating the association between food and nutrient intake and dietary supplement use in Japanese children, although one study examined this association in Japanese adults^(43)^. A nationwide study in Japanese adults revealed that the intake of protein, calcium, iron, vitamin A, folate and vitamin C was lower in dietary supplement users than in non-users^(43)^, which is comparable to the data on meals consumed outside of school in the present study. Discrepancies in findings between studies conducted in Japan and those conducted in other countries may be due to cultural differences in dietary intake and dietary supplement use.

The finding that dietary supplement users consumed less healthy diets than non-users may be explained by the ‘compensation hypothesis’^(42,44–46)^, which suggests that individuals use dietary supplements to compensate for an unhealthy diet. The use of dietary supplements by children may reflect their guardians’ belief that dietary supplements such as multivitamins are a good substitute for fruits and vegetables, based on the rich vitamin and mineral content of these foods. Folate and vitamin C, which were less frequently consumed in dietary supplement users than non-users in the present study, were often used as the nutrient content of vitamins and minerals in dietary supplements. Guardians may give children vitamin and mineral supplements to compensate for dietary gaps, including low intake of fruits and vegetables.

The present study also revealed that while dietary supplement users had a lower intake of fruits and vegetables in meals consumed outside of school, their intake in school lunches was comparable to that of non-users. School lunches are unlikely to reflect the home food environment because Japanese elementary and junior high schools provide school lunches that are high in fruit and vegetable content to all students. Rather, dietary intake at school lunches likely reflects children's food preferences and attitudes toward nutrition. Our findings indicate that familial characteristics may influence dietary supplement use in children.

Extensive evidence indicates that the home food environment is a major determinant of fruit and vegetable intake^(32)^. Therefore, we examined the association between dietary supplement use and well-known indicators of the home food environment, namely, the accessibility, quality, variety and affordability of fruits and vegetables^(30–32)^. Dietary supplement users reported lower accessibility to and satisfaction with the quality of fruits and vegetables available, even though the guardians of all dietary supplement users reported the intention to provide their children with fruits and vegetables. Multivariate regression analyses revealed that subjective accessibility to fruits and vegetables attenuated the association between dietary supplement use and fruit and vegetable intake in children, suggesting that home food availability partly mediated the association between dietary supplement use and fruit and vegetable intake. This implies that although guardians of dietary supplement users are concerned about their children's diet, they may experience barriers towards providing their children with fruits and vegetables.

Some limitations of the present study should be acknowledged. First, the study participants were not randomly selected due to the feasibility of administering school surveys. Nevertheless, the heights and weights of children in the present study were comparable to those of children of the corresponding age and sex based on the national database of health check-ups of Japanese elementary and junior high school children^(3,47)^. Second, individual-level information on maternal education and household income could not be obtained in the present study, as such confidential information on families is challenging to obtain in school surveys. This might lead to inadequate adjustment for socioeconomic status in the association between dietary supplement use and food intake. However, our results showed that users of dietary supplements had a significantly lower intake of fruits and vegetables. Previous studies have shown that dietary supplement users have significantly higher socioeconomic status^(7,15)^, and higher socioeconomic status is associated with a higher intake of fruits and vegetables^(34)^. Therefore, the effect of imprecise socioeconomic status may be minimal. Third, small differences might not be detected in food and nutrient intake between users and non-users of dietary supplements due to the small number of children who used dietary supplements. Fourth, because there is no Japanese database of reliable dietary supplements, we did not calculate nutrient intake from supplements.

In conclusion, the present study demonstrated that dietary supplement use in children was associated with a lower mean intake of fruits and vegetables. The findings of the present study differ from those of previous studies conducted in other countries, suggesting that the dietary characteristics of Japanese supplement users may differ to those in different countries. Further studies are warranted to confirm these results and to investigate whether appropriate dietary supplements are used to compensate for unhealthy diets in children.

## Supplementary material

For supplementary material accompanying this paper visit https://doi.org/10.1017/jns.2021.96. click here to view supplementary material
