# Supplementary material for: Food and nutrient intake in dietary supplement users: a nationwide school-based study in Japan
Source: J Nutr Sci. 2022 Apr 22;11:e29. doi: 10.1017/jns.2021.96 (PMC9066313; doi:10.1017/jns.2021.96)
Supplement: Supplementary file 1 [file jnssup.zip › S2048679021000963sup001.pdf]

RETURN FORM: A hand-signed copy of this LTP must be returned by email to:
jns.edoffice@cambridge.org

NonEx_LTP_Proprietor_05

CAMBRIDGE UNIVERSITY PRESS

NON-EXCLUSIVE GOLD OPEN ACCESS LICENCE TO PUBLISH ("LTP")

This LTP records the terms under which the article specified below will be published in JOURNAL OF NUTRITIONAL SCIENCE (JNS) (the "Journal"). The Journal is exclusively published by the Chancellor, Masters, and Scholars of the University of Cambridge acting through its department Cambridge University Press of University Printing House, Shaftesbury Road, Cambridge CB2 8BS, UK (the "Publisher"). The Journal is owned by THE NUTRITION SOCIETY OF 10 CAMBRIDGE COURT, 210 SHEPHERDS BUSH ROAD, LONDON, W6 7NJ (the "Proprietor").

THE ARTICLE	*Please insert the full title of the article below.*
Article Title*:	Food and nutrient intake in dietary supplement users: a nationwide school-based study in Japan (the "Contribution")

This LTP is for use when the employer(s) of one or more authors own the copyright in their employee's contributions. Each contributing author must be listed on and sign this LTP. Where applicable (that is, where an employer is the copyright owner), an authorised representative of a contributing author's employer should also sign in the relevant box to indicate acceptance of the terms.
Please insert the details of one contributing author per "Author Details and Signatures" box below. Additional authors should be added to the sheet provided at the end of this LTP.

AUTHOR DETAILS AND SIGNATURE(S)			
Full Legal Name*:	Kazue Ishitsuka ("Author")		
Pen or Preferred Name:			
Email Address*:	ishitsuka@ncchd.go.jp	Country of Residence*:	
Signature of Author*:	[illegible]	Date:	October. 14, 2021
Copyright Owner*: *(please tick the appropriate box)*	☑ I own the copyright in (my contributions to) the Contribution, and agree to the terms of this LTP by signing above.<br>☐ My employer owns the copyright in (my contributions to) the Contribution, and a representative of my employer will agree to the terms of this LTP by signing below. This option applies to all types of government employee except U.S Government employees (see below).<br>☐ I am employed by the U.S Government and my employer owns the copyright in my contributions to the Contribution in all jurisdictions outside the U.S. (My contributions to) the Contribution are/is not subject to copyright protection within the U.S. A representative of my employer will agree to the terms of this LTP by signing below.		
Name of Employer *(if applicable)*:	("Employer")		
Name of Employer's authorised signatory *(if applicable)*:			
Signature of Employer's authorised signatory:		Date:	

AUTHOR DETAILS AND SIGNATURE(S)			
Full Legal Name*:	Satoshi Sasaki ("Author")		
Pen or Preferred Name:			
Email Address*:	stssasak@m.u-tokyo.ac.jp	Country of Residence*:	
Signature of Author*:	[illegible]	Date:	October. 14. 2021
Copyright Owner*: *(please tick the appropriate box)*	☑ I own the copyright in (my contributions to) the Contribution, and agree to the terms of this LTP by signing above.<br>☐ My employer owns the copyright in (my contributions to) the Contribution, and a representative of my employer will agree to the terms of this LTP by signing below. This option applies to all types of government employee except U.S Government employees (see below).<br>☐ I am employed by the U.S Government and my employer owns the copyright in my contributions to the Contribution in all jurisdictions outside the U.S. (My contributions to) the Contribution are/is not subject to copyright protection within the U.S. A representative of my employer will agree to the terms of this LTP by signing below.		
Name of Employer *(if applicable)*:	("Employer")		
Name of Employer's authorised signatory *(if applicable)*:			
Signature of Employer's authorised signatory:		Date:	

Additional authors should be added to the sheet provided at the end of this LTP.

CAMBRIDGE EMPLOYEE *You must check this box and enter details, if applicable.*	☐ One or more Authors are employed by Cambridge University Press or are related to a Cambridge University Press employee. Please provide names and describe the relationship(s):

GOLD OPEN ACCESS	*Decide how the Contribution will be accessed by readers*
CHOICE OF LICENCE	By entering into this LTP the Copyright Owner (defined in Clause 1.2) agrees that the VoR (defined in Clause 2.1.2) will be published on a Gold Open Access basis, under the terms of the Creative Commons licence selected below and subject to payment of an APC (defined in Clause 3.5).<br>Indicate, by checking one box below, which Creative Commons licence the VoR should be published under:

PLEASE NOTE: Amended/alternative versions of this LTP will not be accepted and may delay or prevent publication

	☐ **CC BY 4.0** (Attribution)<br>☐ **CC BY NC SA 4.0** (Attribution - Non-commercial - Share Alike)<br>☐ **CC BY NC ND 4.0** (Attribution - Non-commercial - No Derivatives)<br>(each a "Creative Commons Licence")<br>➢ **Please be mindful of the requirements of any funding body/ies (if applicable) when selecting a licence.**<br>➢ More details about Creative Commons Licences: https://creativecommons.org/licenses/<br>➢ If a licence is not selected, the Contribution will be published under the CC BY 4.0 licence

**SUPPLEMENTARY MATERIALS**	*Identify any additional materials to be published in association with the Contribution*
If the Author(s) intend to submit or upload any additional materials for online publication in association with the Contribution, please indicate by checking the applicable boxes in this section.	☐ **NO**, Supplementary Materials will **not** be submitted or uploaded in connection with the Contribution by the Author(s).<br>☐ **YES**, Supplementary Materials which have been entirely created by the Author(s) ("Original SM") will be submitted to the Publisher for publication/uploading on the same terms as the Creative Commons Licence selected.<br>☐ **YES**, Supplementary Materials which contain third-party materials ("Third-party SM") will be submitted to the Publisher for publication/uploading on the same terms as the Creative Commons Licence selected. *(Only check this box if all third-party materials can be published under the Creative Commons Licence terms.)*<br>☐ **YES**, Supplementary Materials which contain third-party materials ("Third-party SM") will be submitted to the Publisher for publication/uploading and the Author(s) shall include a prominent notice stating the licence terms under which those additional materials can be made available.<br>(the "Supplementary Material")

**COPYRIGHT NOTICE**	*If no preferred or suggested copyright notice is indicated below, the copyright notice will identify the Author(s) and/or the Employer(s), as applicable, in accordance with the Publisher's standard practice.*
**Preferred Citation**	*Insert any preferred citation here*
**Use Suggested Citation for U.S Government works?**	☐ **YES**, the Contribution should be accompanied by the following suggested citation:<br>"© [U.S Government Dept] [20XX] outside of the United States of America. As a work owned by the United States Government, this Contribution is not subject to copyright within the United States. Outside of the United States, Cambridge University Press is the non-exclusively licensed publisher of the Contribution."

1 STANDARD TERMS AND CONDITIONS

1.1 Each Author listed in this LTP shall be defined, individually and collectively, as the "**Author(s)**". Each Employer listed in this LTP shall be defined, individually and collectively, as the "**Employer(s)**".

1.2 The Author(s) and the Employer(s) who are identified as the copyright owners in this LTP (together the "**Copyright Owner**") hereby agree to be bound by the following terms and conditions:

2 LICENCE

2.1 The term "**Contribution**" means the article written by the Author(s) as identified on page one of this LTP and includes, without exception, all the following versions of the article:

2.1.1 **Accepted Manuscript ("AM")**: the version of the Contribution that has been accepted for publication. This version may include revisions resulting from peer review but may be subject to further editorial input by the Publisher.

2.1.2 **Version of Record ("VoR")**: the version of the Contribution that is formally published in the Journal. This includes any '*FirstView* article' that is formally identified as being published before the compilation of a volume or issue as long as it is citable via a permanent identifying Digital Object Identifier ("**DOI**"). This does not include any 'early release article' that has not yet been fixed by processes that are still to be applied, such as copy-editing, proof corrections, layout, and typesetting. The VoR includes any corrected or enhanced VoR.

2.2 The term "**Supplementary Material**" means any additional written or illustrative materials submitted or uploaded to the Journal by the Author(s) for publication in connection with the Contribution. Supplementary Material does not form part of the Contribution and will be made available in association with the Contribution in online format only. Supplementary Material may be original content created by the Author(s) ("**Original SM**") or it may be third-party material sourced and cleared in accordance with Clause 5 below, by the Author(s) ("**Third-party SM**").

2.3 If the U.S Government has been identified as the Employer of an Author or Authors, then the Copyright Owner acknowledges that the Contribution is not protected by copyright within the territories of the U.S and that the U.S Government is a joint-owner in relation to any copyright protection which applies to the Contribution outside of the territories of the U.S.

2.4 If the UK, or other applicable commonwealth government, has been identified as the Employer of an Author or Authors, then the Parties acknowledge that Crown Copyright exists in the Work and shall be acknowledged in the Contribution.

2.5 In consideration of publication of the Contribution, the Copyright Owner hereby grants to the Proprietor:

2.5.1 a non-exclusive licence to publish, reproduce, distribute, and sell the Contribution, or any part of it, and any Supplementary Material in all forms and media and in all languages throughout the world, whether print, digital / electronic, whether now known or hereinafter invented, and to grant sublicences of all translation and subsidiary rights; and

2.5.2 a non-exclusive licence to exploit all other rights in the nature of copyright, including rental, lending and database rights and all other publishing and print on demand rights in the Contribution and any Supplementary Material.

2.6 The licences described in Clause 2.5 above shall, throughout this LTP, be referred to collectively as the "**Licence**".

2.7 The Licence shall commence upon the Proprietor's formal acceptance to publish the Contribution and shall endure for the legal term of copyright in the Contribution.

2.8 The Author(s) hereby assert(s) his/her/their moral right always to be identified as the author(s) of the Contribution in accordance with the provisions of the UK Copyright, Designs and Patents Act 1988.

3 GOLD OPEN ACCESS

3.1 The Publisher will publish the VoR as part of the Journal, on a Gold Open Access basis.

3.2 "**Open Access**" means content which is distributed in digital format to the end-user without charge. Open Access *may* confer certain rights in the content to the end-user, including the ability to re-distribute it or to create derivative works from it.

3.3 For details of the Publisher's Open Access policies please follow the relevant hyperlinks at: **www.cambridge.org/openaccess.**

3.4 "**Gold Open Access**" means the Publisher will make the VoR freely accessible on the Publisher's website under the terms of the Creative Commons Licence chosen by the Author(s) in this LTP.

3.5 "**Article Processing Charge**" or "**APC**" means the payment by the Copyright Owner, or a third-party on the behalf of the Copyright Owner, to the Publisher in consideration for the publication of the VoR on the Publisher's website under the terms of this LTP. The VoR will not be published until the APC is paid in full, unless the Publisher, at its sole discretion, waives or discounts the APC or the Contribution is covered by alternative funding provided in lieu of an APC under a separate agreement between the Publisher and a relevant institution or funding body.

3.6 Where applicable, a separate invoice shall be issued to the appropriate entity for the payment of the APC and the Author(s) is/are responsible for providing the Publisher with sufficient details to issue the invoice.

3.7 For the avoidance of doubt, publication of the VoR under the CC BY NC SA or CC BY NC ND Creative Commons Licences expressly does **not** permit any commercial reuse without permission from the Publisher. The table below summarises when and where the VoR can be posted, depending on the Creative Commons Licence chosen by the Author(s):

Version & Creative Commons Licence	Author(s)'s personal webpage	Author(s)'s department / institutional repository	Non - commercial subject repository	Commercial repository / social media
VoR (under CC BY)	From first publication	From first publication	From first publication	From first publication
VoR (under CC BY NC SA or CC BY NC ND)	From first publication	From first publication	From first publication	Not permitted

3.8 For the avoidance of doubt, any type of reuse of the VoR which is not provided for under the terms of this Clause 3 and by the chosen Creative Commons Licence shall remain subject to the Publisher's permission.

4 UNDERTAKINGS AND REPRESENTATIONS

4.1 Notwithstanding the Licence, the Copyright Owner agrees and undertakes that, at the time of execution of this LTP, and thereafter until the publication of the VoR, the Contribution or any prior version of it in whole or in part, is not currently under and will not be submitted for consideration for publication in any peer-reviewed scholarly journal.

4.2 The Copyright Owner hereby undertakes and represents that:

4.2.1 each named Author and/or Employer has full authority and power to agree to this LTP;

4.2.2 the Contribution is original and has not been previously published in whole or in part;

4.2.3 the Contribution and any Supplementary Material contain nothing that infringes any existing copyright or licence or any other intellectual property right of any third-party;

4.2.4 the Contribution and any Supplementary Material contain nothing that breaches a duty of confidentiality or discloses any private or personal information of any person without that person's written consent;

4.2.5 all statements contained in the Contribution and any Original SM purporting to be facts are true and any formula, instruction or equivalent contained therein will not, if followed accurately, cause any injury or damage to the user;

4.2.6 the Contribution and any Supplementary Material do not contain any libellous or otherwise unlawful material, or any material which would harm the reputation of the Publisher or the Proprietor;

4.2.7 there are no actual or apparent conflicts of interest connected to the Contribution that have not previously been declared. A conflict of interest is understood to exist if an interest (financial or otherwise) exerts or appears to exert undue influence on the analysis or conclusions in the Contribution, the choice of subject matter, or in any other way that impedes or appears to impede the Author(s)'s objectivity or independence.

4.3 In the event that the Copyright Owner is in breach of any of these undertakings the Proprietor and/or the Publisher shall have the right to cease making the Contribution and/or any Supplementary Material available and/or to require that the Author(s) make any necessary revisions to the Contribution and/or any Supplementary Material (including any factual information). Any such revisions shall be governed by this LTP.

5 THIRD-PARTY MATERIALS

5.1 The Copyright Owner further confirms that for (i) any Third-party SM and (ii) any other third-party material (including but not limited to textual, illustrative, audio and video content) within the Contribution:

5.1.1 licences to re-use said content throughout the world in all languages and in all forms and media have or will be obtained from the rights-holders;

5.1.2 appropriate acknowledgement to the original source of all such materials has been made; and

5.1.3 in the case of audio/video material, appropriate release forms have been obtained from the individual(s) whose likenesses are represented in the Contribution and/or Third-party SM, as applicable.

5.2 Copies of all licences and/or release documentation acquired in accordance with Clause 5.1 above will, on request, be forwarded to the Journal's editor prior to publication of the Contribution.

5.3 The Author(s) must ensure that all third-party permission obtained under Clause 5.1 above allows third-party material to be included in a work licensed under the Creative Commons Licence.

6 MISCELLANEOUS

6.1 The Publisher cooperates with various copyright licensing schemes which allow material to be photocopied within agreed restraints (e.g. the Copyright Clearance Center in the U.S and the Copyright Licensing Agency in the UK). Any proceeds received by the Publisher from such licences, together with any proceeds resulting from sales of subsidiary rights in the Contribution, shall be used by the Publisher to support the continuing publication of its academic works.

6.2 The information contained in this LTP will be held for record-keeping purposes. The name(s) of the Author(s) and any Employer(s) may be reproduced in the Journal and provided to print and online indexing and abstracting services and bibliographic databases. The Proprietor and the Publisher comply with applicable data protection and privacy laws in the collection, retention, storage, and use of personal data.

7 ENTIRE AGREEMENT

7.1 This LTP is made between, and contains the entire agreement between, the Proprietor and the Copyright Owner concerning the Contribution and supersedes all related prior agreements, arrangements and understandings (whether written or oral). No addition to or modification of any provision of this LTP shall be binding unless it is in writing and signed on behalf of the Proprietor and the Copyright Owner.

7.2 The Copyright Owner acknowledges and agrees that the Proprietor is responsible, at its discretion, for appointing 'publisher(s)' to fulfil all or part of the Proprietor's and Publisher's obligations under this LTP, provided that any new 'publisher' appointed by the Proprietor shall comply with the terms of this LTP.

7.3 This LTP is governed by the law of England and Wales and is subject to the exclusive jurisdiction of the English courts.

7.4 This LTP may be executed in any number of counterparts, each of which when executed shall be deemed to be a duplicate original, but all of which, taken together, shall constitute one and the same agreement. The LTP shall not take effect until each Author and Employer has executed and delivered its counterpart to the Publisher or the Proprietor.

 PLEASE NOTE: Amended/alternative versions of this LTP will not be accepted and may delay or prevent publication

ADDITIONAL AUTHOR(S)

Each contributing author must be listed on and sign this LTP. Where applicable (that is, where an employer is the copyright owner), an authorised representative of a contributing author's employer should also sign in the relevant box to indicate acceptance of the terms.
Please insert the details of one contributing author per "Author Details and Signatures" box below. If necessary, please photocopy this page and attach it to your LTP.

AUTHOR DETAILS AND SIGNATURE(S)			
**Full Legal Name*:**	Keiko Asakura ("Author")		
**Pen or Preferred Name:**			
**Email Address*:**	keiko.asakura@med.toho-u.ac.jp	**Country of Residence*:**	Japan
**Signature of Author*:**	Keiko Asakura	**Date:**	Oct. 14, 2021.
**Copyright Owner*:** *(please tick the appropriate box)*	☑ I own the copyright in (my contributions to) the Contribution, and agree to the terms of this LTP by signing above.<br>☐ My employer owns the copyright in (my contributions to) the Contribution, and a representative of my employer will agree to the terms of this LTP by signing below. This option applies to all types of government employee except U.S Government employees (see below).<br>☐ I am employed by the U.S Government and my employer owns the copyright in my contributions to the Contribution in all jurisdictions outside the U.S. (My contributions to) the Contribution are/is not subject to copyright protection within the U.S. A representative of my employer will agree to the terms of this LTP by signing below.		
**Name of Employer** *(if applicable)*:	("Employer")		
**Name of Employer's authorised signatory** *(if applicable)*:			
**Signature of Employer's authorised signatory:**		**Date:**	

AUTHOR DETAILS AND SIGNATURE(S)			
**Full Legal Name*:**	("Author")		
**Pen or Preferred Name:**			
**Email Address*:**		**Country of Residence*:**	
**Signature of Author*:**		**Date:**	
**Copyright Owner*:** *(please tick the appropriate box)*	☐ I own the copyright in (my contributions to) the Contribution, and agree to the terms of this LTP by signing above.<br>☐ My employer owns the copyright in (my contributions to) the Contribution, and a representative of my employer will agree to the terms of this LTP by signing below. This option applies to all types of government employee except U.S Government employees (see below).<br>☐ I am employed by the U.S Government and my employer owns the copyright in my contributions to the Contribution in all jurisdictions outside the U.S. (My contributions to) the Contribution are/is not subject to copyright protection within the U.S. A representative of my employer will agree to the terms of this LTP by signing below.		
**Name of Employer** *(if applicable)*:	("Employer")		
**Name of Employer's authorised signatory** *(if applicable)*:			
**Signature of Employer's authorised signatory:**		**Date:**	

AUTHOR DETAILS AND SIGNATURE(S)			
**Full Legal Name*:**	("Author")		
**Pen or Preferred Name:**			
**Email Address*:**		**Country of Residence*:**	
**Signature of Author*:**		**Date:**	
**Copyright Owner*:** *(please tick the appropriate box)*	☐ I own the copyright in (my contributions to) the Contribution, and agree to the terms of this LTP by signing above.<br>☐ My employer owns the copyright in (my contributions to) the Contribution, and a representative of my employer will agree to the terms of this LTP by signing below. This option applies to all types of government employee except U.S Government employees (see below).<br>☐ I am employed by the U.S Government and my employer owns the copyright in my contributions to the Contribution in all jurisdictions outside the U.S. (My contributions to) the Contribution are/is not subject to copyright protection within the U.S. A representative of my employer will agree to the terms of this LTP by signing below.		
**Name of Employer** *(if applicable)*:	("Employer")		
**Name of Employer's authorised signatory** *(if applicable)*:			
**Signature of Employer's authorised signatory:**		**Date:**	

PLEASE NOTE: Amended/alternative versions of this LTP will not be accepted and may delay or prevent publication
