# Supplementary material for: Food and nutrient intake in dietary supplement users: a nationwide school-based study in Japan
Source: J Nutr Sci. 2022 Apr 22;11:e29. doi: 10.1017/jns.2021.96 (PMC9066313; doi:10.1017/jns.2021.96)
Supplement: Supplementary file 1 [file jnssup.zip › S2048679021000963sup002.docx]

Supplementary Table 1. Food groups

|  | Example food items | Item number in the Standard Tables of Food Composition in Japan 2015 ^(19)^ |
| --- | --- | --- |
| Cereals | White rice, brown rice, bread, noodles | 1001-1166 |
| Potatoes | Potatoes, sweet potatoes | 2001-2062 |
| Confectionaries | Japanese confectioneries, snacks, cakes, jam | 3001-3023, 15001-15140 |
| Nuts and pulses | Almonds, peanuts, soybeans, soy products | 4001-4094, 5001-5037 |
| Fruits and vegetables | Green vegetables, white vegetables, mushrooms, seaweed, fruit | 6001-6326, 7001-7155, 8001-8036, 9001-9047 |
| Oils | Plant oils, mayonnaise, butters | 10001-10388 |
| Teas | Black tea, green tea, oolong tea | 11001-11244, 12001-12020 |
| Fish | Horse mackerel, salmon, tuna, shellfish, shrimp | 13001-13050 |
| Meats and eggs | Beef, pork, chicken, ham, bacon | 14001-14031 |
| Dairy products | Milk, yogurt, cheese | 16033-16049, 16051, 16055 |
| Seasonings | Soy sauce, salt, vinegar, miso, soup stock | 16052-16054 |
